# An integrative association method for omics data based on a modified Fisher’s method with application to childhood asthma

**DOI:** 10.1371/journal.pgen.1008142

**Published:** 2019-05-07

**Authors:** Qi Yan, Nianjun Liu, Erick Forno, Glorisa Canino, Juan C. Celedón, Wei Chen

**Affiliations:** 1 Division of Pediatric Pulmonary Medicine, UPMC Children’s Hospital of Pittsburgh, University of Pittsburgh, Pittsburgh, PA; 2 Department of Epidemiology and Biostatistics, School of Public Health, Indiana University Bloomington, Bloomington, IN; 3 Behavioral Sciences Research Institute, University of Puerto Rico, San Juan, PR; 4 Department of Biostatistics, Graduate School of Public Health, University of Pittsburgh, Pittsburgh, PA; 5 Department of Human Genetics, Graduate School of Public Health, University of Pittsburgh, PA; Case Western Reserve University, UNITED STATES

## Abstract

The development of high-throughput biotechnologies allows the collection of omics data to study the biological mechanisms underlying complex diseases at different levels, such as genomics, epigenomics, and transcriptomics. However, each technology is designed to collect a specific type of omics data. Thus, the association between a disease and one type of omics data is usually tested individually, but this strategy is suboptimal. To better articulate biological processes and increase the consistency of variant identification, omics data from various platforms need to be integrated. In this report, we introduce an approach that uses a modified Fisher’s method (denoted as Omnibus-Fisher) to combine separate *p*-values of association testing for a trait and SNPs, DNA methylation markers, and RNA sequencing, calculated by kernel machine regression into an overall gene-level *p*-value to account for correlation between omics data. To consider all possible disease models, we extend Omnibus-Fisher to an optimal test by using perturbations. In our simulations, a usual Fisher’s method has inflated type I error rates when directly applied to correlated omics data. In contrast, Omnibus-Fisher preserves the expected type I error rates. Moreover, Omnibus-Fisher has increased power compared to its optimal version when the true disease model involves all types of omics data. On the other hand, the optimal Omnibus-Fisher is more powerful than its regular version when only one type of data is causal. Finally, we illustrate our proposed method by analyzing whole-genome genotyping, DNA methylation data, and RNA sequencing data from a study of childhood asthma in Puerto Ricans.

## Author summary

In this research, we developed a statistical approach using a modified Fisher’s method (denoted as Omnibus-Fisher) to combine separate *p*-values of association testing for a trait and SNPs, DNA methylation markers, and RNA sequencing, calculated by kernel machine regression into an overall gene-level *p*-value to account for correlation between omics data. We further extended the method to an optimal version in order to consider all possible disease models.

## Introduction

Because of major advances in high-throughput biotechnologies, large amounts of omics data have been collected to study the biological mechanisms underlying complex diseases at different levels, such as genomics, epigenomics, and transcriptomics. Such different types of omics data can help us understand a disease from several perspectives. However, each of the arrays or sequencing technologies is designed to collect a specific type of omics data, such as SNPs, DNA methylation markers, and RNA sequencing. Thus, the association between a complex disease and one type of omics data is usually tested individually, but this strategy is suboptimal and has some disadvantages. Researchers often find that only a small proportion of disease variation can be explained by one type of omics (e.g., genetic) data, leading to “missing heritability” [1]. Moreover, molecular variants identified by different studies usually suffer from poor reproducibility [2, 3]. Most importantly, only partial information is used for each individual analysis. Therefore, in order to better characterize biological processes and increase the consistency of variant identification, omics data from separate platforms need to be integrated and analyzed. Integrating information from different biological datasets has the potential to yield better insight into causal mechanisms of complex diseases than that from individual omics datasets.

Although integrative analysis of omics data is clearly needed, the complexity of disease mechanisms, the large number of collected molecular variables, and relatively small datasets can make such analysis quite challenging. Bersanelli et al. [4] summarized a list of existing statistical approaches for integrative analysis. When developing integrative analysis methods, two inevitable issues are: 1. handling a large number of variables and 2. dealing with data from relatively small studies. In genetic studies, to handle a large number of genetic variants in a gene, gene-based approaches [5–15] have been developed to evaluate the joint effects of genetic variants in the same gene on the disease of interest. Of the existing methods, the sequence kernel-machine-based associations test (SKAT) [16, 17] is a powerful, flexible, and computationally efficient test. In this kernel machine (KM) approach, the test statistic follows a mixture of chi-square distributions, and thus *p*-values can be computed analytically and quickly without using resampling techniques. Although gene-based tests were originally developed for genetic studies, the same concept can be applied to studies of multi-omics data. Another issue is small sample size, especially for epigenomic or transcriptomic data. For example, large-scale genome wide association studies (GWASs) have been widely conducted for genetic studies for many years, so researchers usually have hundreds or thousands of genotyped samples. However, genome- wide methylation studies are more recent, and thus researchers often have a small number of samples. Moreover, incomplete samples may be wasted when using methods requiring complete samples (e.g., methods incorporating multi-omics data variables into one regression model). In this scenario, methods combining multiple *p*-values can be applied to make full use of data. For example, the *p*-values for association testing of a disease and SNPs, DNA methylation markers, and RNA sequencing data are be calculated separately, and then these separate *p*-values can be appropriately combined into one final *p*-value.

In order to test for overall gene-level significance, we here present an approach to use a modified Fisher’s method (denoted as Omnibus-Fisher) to combine separate *p*-values for association testing of a disease or trait and SNPs, methylation markers, and RNA sequencing data calculated by KM regression into an overall gene-level *p*-value accounting for correlation between omics data. This method can be applied to either samples with all three types of omics data or samples with one or two types. To account for all possible disease models, we further extend the modified Fisher’s method to an optimal test by using perturbations. In our simulation studies, we show that a usual Fisher’s method has inflated type I error rates when directly applied to correlated omics data. In contrast, our Omnibus-Fisher test preserves the expected type I error rates when employed in correlated omics data. Moreover, the Omnibus-Fisher method has increased power compared to its optimal version when the true disease model involves all of SNPs, methylation markers, and RNA sequencing data. On the other hand, the optimal Omnibus-Fisher method is more powerful than its regular version when only one type of data is causal. Finally, we illustrate our proposed methodology by analyzing whole-genome genotyping, DNA methylation, and RNA sequencing data from a study of childhood asthma in Puerto Ricans.

## Results

### Simulation of the Type I error rate

**Fig 1:**
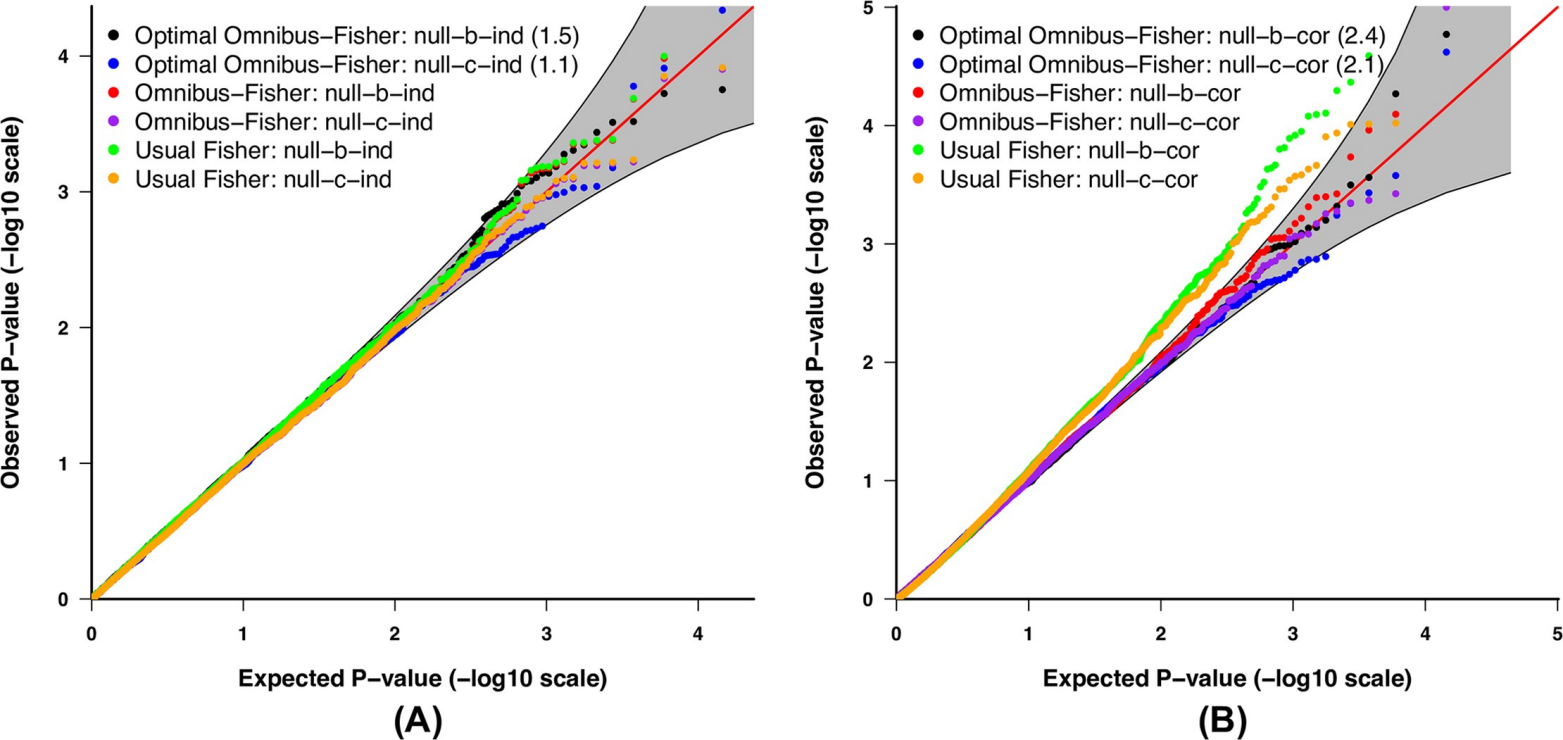
QQ plot of the *p*-values. A 95% pointwise confidence band (gray area) was computed under the assumption that the *p*-values were drawn independently from a uniform [0, 1] distribution. (A) Independent G, M and E; and (B) G and E correlated.

When applied to samples with independent SNPs (G), methylation markers (M), and RNA sequencing data (E), all of the methods used (i.e., the Fisher’s methods with and without considering *p*-value covariance [Omnibus-Fisher and usual Fisher], and the optimal test with *p*-values from Omnibus-Fisher as inputs [optimal Omnibus-Fisher]) had empirical Type I error rates close to the nominal level (**Fig 1A** and **Table 1**). When the usual Fisher’s method without considering covariance was applied to G and E correlated data, the Type I error rate was inflated (**Fig 1B** and **Table 1**). In contrast, the optimal and regular Omnibus-Fisher methods with considering covariance retained the desired Type I error rates as evidenced by the patterns observed in the QQ plots shown in **Fig 1B** and **Table 1**. Similar results were observed when extending to 100,000 datasets for evaluation (S1 Table).

**Table 1:** Simulated Type I error rates based on 10,000 datasets.

Significance level	Optimal Omnibus-Fisher	Omnibus-Fisher	Usual Fisher
*Independent G*, *M and E with binary traits*			
0.05	0.0521	0.0516	0.0522
0.01	0.0100	0.0109	0.0108
0.001	0.0015	0.0015	0.0015
*Independent G*, *M and E with continuous traits*			
0.05	0.0483	0.0472	0.0478
0.01	0.0088	0.0095	0.0095
0.001	0.0007	0.0009	0.0009
*G and E correlated with binary traits*			
0.05	0.0488	0.0526	0.0675
0.01	0.0089	0.0107	0.0161
0.001	0.0010	0.0015	0.0029
*G and E correlated with continuous traits*			
0.05	0.0525	0.0509	0.0655
0.01	0.0093	0.0096	0.0166
0.001	0.0005	0.0011	0.0029

### Statistical power comparison

**Fig 2:**
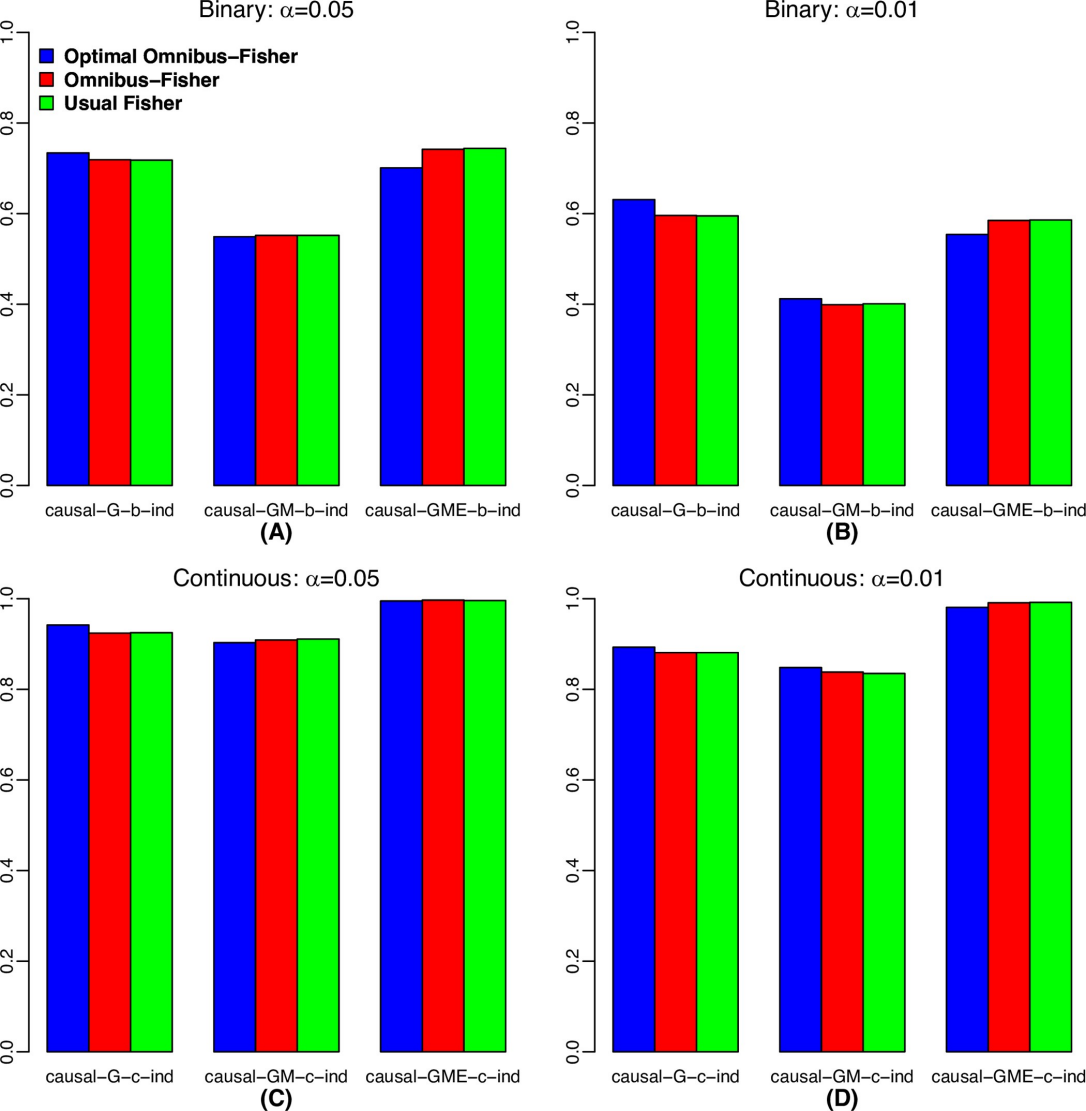
Power comparison for the scenario of independent G, M and E. (A) Binary trait at α = 0.05; (B) Binary trait at α = 0.01; (C) Continuous trait at α = 0.05; and (D) Continuous trait at α = 0.01.

**Fig 3:**
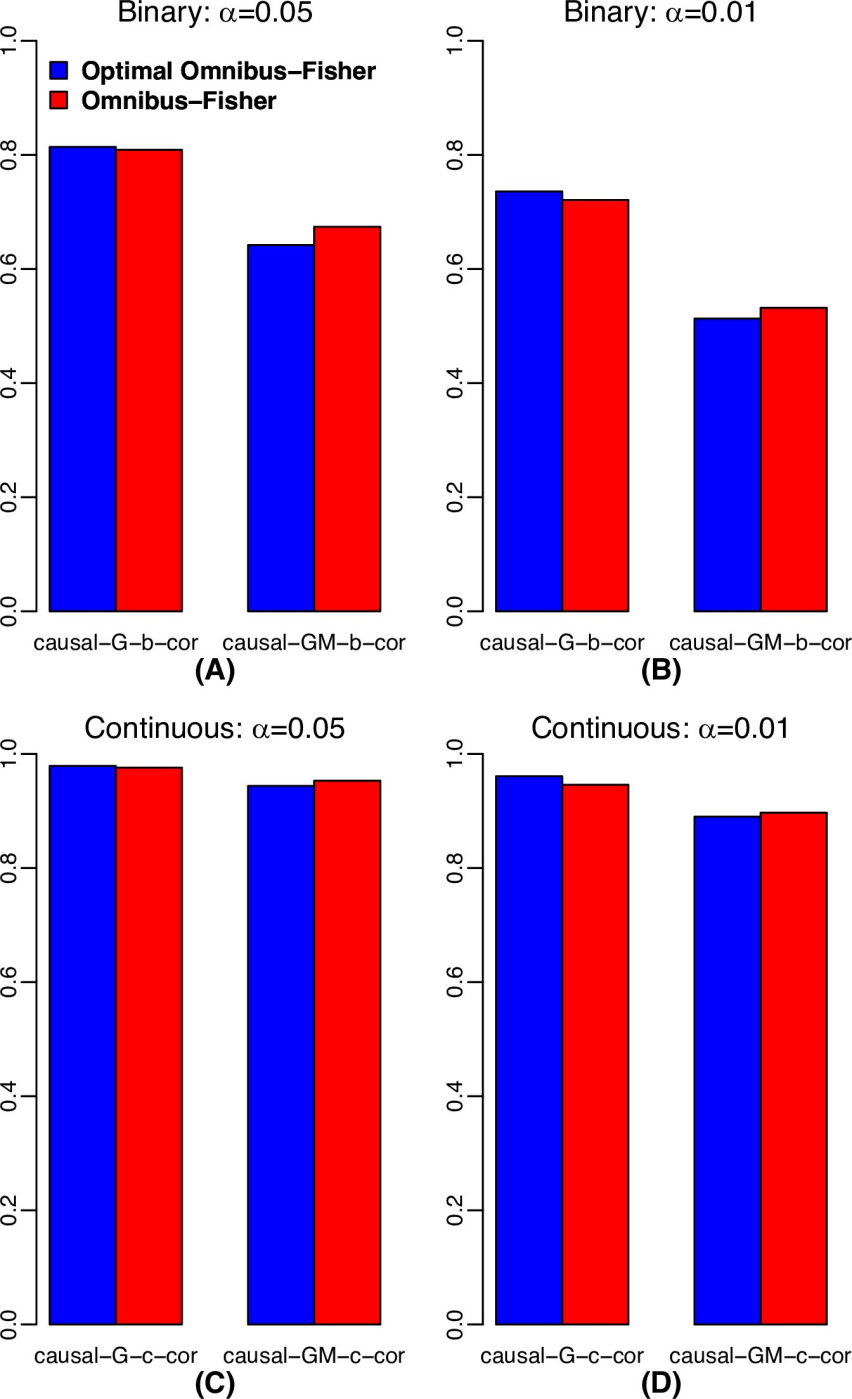
Power comparison for the scenario of G and E correlated. (A) Binary trait at α = 0.05; (B) Binary trait at α = 0.01; (C) Continuous trait at α = 0.05; and (D) Continuous trait at α = 0.01.

When we compared the power of the statistics on the samples with independent G, M and E (**Fig 2**), the power of optimal Omnibus-Fisher was consistent higher than that of the regular Omnibus-Fisher method when G was the only causal factor, but when G, M and E were all causal factors, the optimal methods had lower power. This was expected because the optimal methods automatically searched for the appropriate disease model; in contrast, the regular Omnibus-Fisher assumed that G, M and E were all causal factors. Thus, when the simulation matched the assumption of the regular version method, they performed better than the optimal version and vice versa. However, when G and M were causal factors, no methods were consistently better than another. Furthermore, similar patterns were observed, when evaluated using the samples with G and E correlated (**Fig 3**). Since the causal SNPs in G were correlated with E, GM causal was equivalent to G, M and E causal. Note that the usual Fisher’s method is not included in **Fig 3** because of its inflated Type I error rate with correlated data.

### Results of childhood asthma in Puerto Ricans

**Fig 4:**
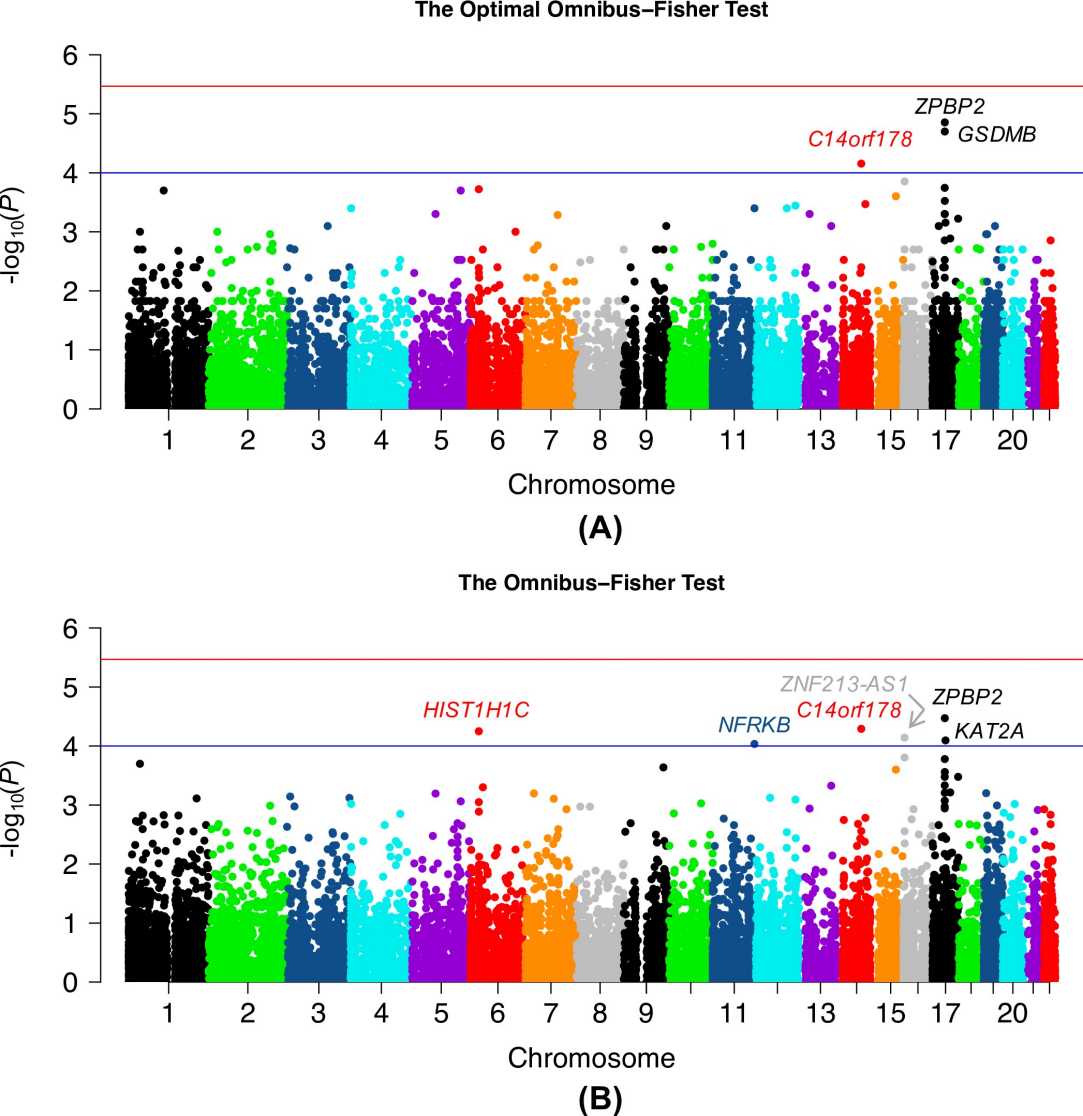
–log_10_(*p*-values) of the association between 14,808 genes and asthma status in WBCs. (A) The optimal Omnibus-Fisher test and (B) the regular Omnibus-Fisher test. The blue line is the suggestive significance level, 1×10^−4^, and the red line is the stringent Bonferroni-corrected significance level, *P* = 3.38×10^−6^.

We used the proposed optimal Omnibus-Fisher statistic and its regular version to analyze the Puerto Rican childhood asthma data from WBCs for associations between asthma status and 14,808 genes with all SNPs, DNA methylation markers, and gene expression, with adjustment for age, gender and first two principal components calculated based genotypes. In addition, batch effect and cell type composition were also adjusted for DNA methylation and RNA sequencing data. We found that *ZPBP2* was the most significant gene from both optimal (*P* = 1.40×10^−5^) and regular (*P* = 3.39×10^−5^) Omnibus-Fisher tests, although it didn’t reach a Bonferroni corrected significance level (*P* = 3.38×10^−6^) (**Fig 4**). The *ZPBP2* region from chromosome 17q21 has been consistently replicated as an asthma-susceptibility locus across diverse ethnic groups [18–28] including Puerto Ricans [29] and this region regulates its gene expression in Puerto Ricans [30]. In a meta-analysis of GWAS in Puerto Ricans [29], the only region associated with asthma was the *ZPBP2* locus and the current genotypic dataset was analyzed as a part of the data. This gene could be served as a positive control in asthma genetic studies. In the optimal Omnibus-Fisher test, the significance of *ZPBP2* as well as *GSDMB* was mainly driven by their genetic effect (*P* = 2.89×10^−6^ for *ZPBP2* and 2.36×10^−6^ for *GSDMB*). Moreover, five additional genes (*KAT2A*, *HIST1H1C*, *NFRKB*, *C14orf178* and *ZNF213-AS1*) were suggestively associated with asthma (*P* < 0.0001 [**Table 2**]) from the regular Omnibus-Fisher test. Of these genes, *KAT2A* had moderate effects for SNPs, DNA methylation, and RNA expression separately, which could be overlooked by a single type of data analysis. The results also indicate that the optimal Omnibus-Fisher test was more powerful than its regular version when the significance was driven by one type of data. Conversely, when statistical significance was driven by two or three types of data, the regular Omnibus-Fisher had overall better power than the optimal version. These observations were generally consistent with the simulation results. Here, the optimal Omnibus-Fisher test does not outperform its regular version that assumes all types of omics data are in the disease model. Since only three types of omics data were analyzed in this study, it was still fine to assume they all were in the disease model. However, when more omics data are analyzed, the optimal test could be more useful than simply assuming all types of data are causal. We additionally output the p-value correlation for each gene across the whole genome (S1 Fig): 1. between SNPs and DNA methylation markers, 2. between SNPs and expression genes, and 3. between DNA methylation markers and expression genes.

**Table 2:** Genes with *P* < 1×10^−4^ from the optimal or regular Omnibus-Fisher tests for the asthma status analysis in WBCs.

*P*-values Genes Chr		Optimal Omnibus-Fisher	Regular Omnibus-Fisher	Gene-level SNP	Gene-level Methylation	Gene-level RNA expression
*ZPBP2*	17	1.40×10^−5^	3.39×10^−5^	2.89×10^−6^	0.1071	0.6728
*GSDMB*	17	2.00×10^−5^	1.67×10^−4^	2.36×10^−6^	0.8816	0.6153
*KAT2A*	17	7.00×10^−4^	8.06×10^−5^	0.0102	0.0047	0.0127
*HIST1H1C*	6	1.90×10^−4^	5.65×10^−5^	0.2343	0.0109	2.00×10^−4^
*NFRKB*	11	4.00×10^−4^	9.25×10^−5^	0.0086	4.76×10^−4^	0.1779
*C14orf178*	14	7.00×10^−5^	5.13×10^−5^	0.1012	2.30×10^−5^	0.1757
*ZNF213-AS1*	16	0.0020	7.25×10^−5^	3.57×10^−4^	0.0688	0.0197

Analysis of the WBC genome-wide data with 1,116 samples and 14,808 genes took ~108.8 hours on a single computing node with a 3 GHz CPU and 4 GB memory. Using a computer cluster with multiple nodes, we anticipate that genome-wide data analysis should be finished within hours using our proposed methods.

## Discussion

In this work, we developed an Omnibus-Fisher statistic using a kernel machine (KM) regression framework, which can be employed to test overall gene-level significance by combining separate *p*-values of association testing for a disease and SNPs, methylation markers, and expression genes, accounting for correlation between omics data. The separate *p*-values are calculated by gene-based KM regression. The gene-based analysis methods can improve power by testing a set of variants jointly and by reducing the multiple testing penalty. In addition, the method using a gene as the unit can easily combine different types of omics data that are mapped to the same gene and thus easily interpret the results. Since we do not know the exact disease model in reality, the extended optimal Omnibus-Fisher test can account for all possible disease models. Moreover, our proposed tests can be applied to either samples with all three types of omics data or with one or two types. In other words, samples with incomplete data can still contribute to the test statistic. The information about whether the different types of omics data are from the sample can also be accounted.

In the simulation studies, we showed that using a usual Fisher’s method on correlated omics data results in an inflated Type I error rate, while the modified Fisher’s method, Omnibus-Fisher, had the correct Type I error rate because it considered the omics data correlation in the model. The Omnibus-Fisher method achieves better power performance compared to its optimal version when the true disease model involves all of SNPs, RNA expressions and DNA methylations. On the other hand, the optimal Omnibus-Fisher method has better power than its regular version assuming all types of data are causal when only one type of data is actually causal. Our real data study also shows that the regular Omnibus-Fisher test has better power than the optimal test, when two or three types of data contribute to the combined *p*-value. Because we only consider three types of omics data in this study, assuming they all are causal could be still acceptable. However, when more omics data are analyzed, we believe that the optimal test would be more powerful for most genes than simply assuming all types of data are causal. Nevertheless, both the optimal and regular Omnibus-Fisher tests are able to detect genes with moderate separate effects, which could be overlooked by single type of data analyses.

Although the optimal Omnibus-Fisher test uses perturbation to consider the correlation between omics data and search for the optimal disease model, the genome-wide data analysis could be completed within hours using multiple CPUs (e.g., one CPU for each chromosome). We adapt a stepwise manner to implement perturbation (e.g., more iterations for smaller *p*-values) so as to save computation times when calculating large *p*-values. Thus, the majority of the computation time is used by genes with small *p*-values. However, if a large number of genes are highly associated with the phenotype, the optimal test may be infeasible due to computational intensity. In such case, the regular test is recommended. Although the regular test also involves resampling technique to calculate covariances between different types of omics data, it only requires a small number of resampling (e.g., the default setting is 200 times).

Our method framework is general and flexible. Both continuous and binary traits for independent samples can be analyzed. Covariates can be easily incorporated into the model and different covariates can be used for different omics data. The regular and optimal version of Omnibus-Fisher algorithms were implemented in R (http://www.r-project.org) and the R package (https://cran.r-project.org/web/packages/OmnibusFisher/index.html) is available.

## Materials and methods

### KM Regression for testing gene-level effects of SNPs, DNA methylation and RNA expression

We used KM regression to calculate the gene-level *p*-values for association testing of a disease and SNPs, methylation markers, and expression genes. First, we test the effect of SNPs. Let there be *n* subjects with *q* genetic variants. The *n* × 1 vector of the continuous trait **y** follows a linear model:

y=Xβ+Gγ+ε,

when the phenotypes are binary, **y** follows a logistic model:

logitP(y=1)=Xβ+Gγ

where **X** is an *n* × *p* covariate matrix, **β** is a *p* × 1 vector containing parameters for the fixed effects (an intercept and *p–* 1 covariates), **G** is an *n* × *q* genotype matrix for the *q* genetic variants of interest where an additive genetic model is assumed (i.e., coded as 0, 1, or 2 representing the copies of minor alleles) for illustration, **γ** is a *q* × 1 vector for the random effects of the *q* genetic variants, and **ε** is an *n* × 1 vector for the random error. The random effect *γ*_*j*_ for variant *j* is assumed to be normally distributed with mean zero and variance *τw*_*j*_; thus, the null hypothesis *H*_*0*_: **γ** = 0 is equivalent to *H*_*0*_: *τ* = 0, which can be tested with a variance component score test [17] in the mixed model. The random variable **ε** is assumed to be normally distributed, and is uncorrelated with **γ**:

γ∼N(0,τW)

$$\varepsilon ∼N(0,\;\sigma_{E}^{2}I),$$

where **W** is a predefined *q* × *q* diagonal weight matrix for each variant and may use **W = I** when lacking of prior information, and $\sigma_{E}^{2}$ is the error variance.

Following the same rationale as in the derivation of the SKAT score statistic [31–33], the test statistic is:

$$Q={\left(y-X\hat{\beta }\right)}^{\prime }GWG^{\prime }(y-X\hat{\beta })/{\hat{\sigma }}_{E}^{2},$$

when phenotypes are continuous, and

$$Q=(y-\hat{\mu }{)}^{\prime }GWG^{\prime }(y-\hat{\mu })$$

when phenotypes are binary, where $\hat{\beta }$ is the vector of estimated fixed effects of covariates under *H*_*0*_ and $\hat{\mu }={logit}^{-1}(X\hat{\beta })$.

Under the null hypothesis, the linear model is **y** = **Xβ** + **ε**, and the estimates are

$$\hat{\Sigma }={\hat{\sigma }}_{E}^{2}I=var(y-X\hat{\beta })I$$

$$\hat{\beta }={\left(X^{\prime }X\right)}^{-1}X^{\prime }y$$

$$P_{0}=I-X{\left(X^{\prime }X\right)}^{-1}X^{\prime };$$

the logistic model is logit *P*(**y** = **1**) = **Xβ**, and the estimates are

$$\hat{\Sigma }=diag(\hat{\mu }∙(1-\hat{\mu }))$$

$$\hat{\beta }={\left(X^{\prime }{\hat{\Sigma }}^{-1}X\right)}^{-1}X^{\prime }{\hat{\Sigma }}^{-1}y$$

$$P_{0}=\hat{\Sigma }-\hat{\Sigma }X{\left(X^{\prime }\hat{\Sigma }X\right)}^{-1}X^{\prime }\hat{\Sigma }.$$

The statistic Q is a quadratic form and follows a mixture of chi-square distributions under *H*_*0*_. Thus,

$$Q∼\sum_{i=1}^{q}\lambda_{i}\chi_{1,i}^{2},$$

where *λ*_*i*_ are the eigenvalues of the matrix ${P_{0}}^{\frac{1}{2}}GWG^{\prime }{P_{0}}^{\frac{1}{2}}$ [34] for both continuous and binary traits. The *p*-values can be calculated by numerical algorithms, such as Davies’ method [35] and Kuonen’s saddlepoint method [36], which are both included in the R package.

Analogously, the gene-level effects of DNA methylation markers and expression genes can be tested by replacing **Gγ** with **Mρ** and **Eη**, **M** is an *n* × *k* matrix for the *k* methylated loci, **ρ** is a *k* × 1 vector for the random effects of the *k* methylated loci, **E** is an *n* × *g* matrix for the RNA expression, and **η** is a *g* × 1 vector for the random effects of the RNA expression. When using microarray platform, multiple probes could map to the same gene and each probe has an expression value, which result in more than one expression value for one gene. Here, *g* is the number of probes for one gene. When using RNA sequencing platform, one gene can always have one expression value (i.e., **E** is an *n* × *1* vector and **η** is a scalar), although it is also possible to obtain the transcript (i.e., isoform) level expression values. The null hypothesis is **ρ** = 0 for testing DNA methylation markers and **η** = 0 for testing expression genes. It is worth to note that all three models can have the same or different null models.

### Modified Fisher’s method (Omnibus-Fisher) for combining gene-level effects of SNPs, DNA methylation and RNA expression

In order to have one single *p*-value to represent the significance of a gene, we propose an approach to test if the trait is associated with any SNP, DNA methylation marker, and RNA sequencing variant. This could help researchers to screen out potentially interesting genes. Thus, after obtaining the three *p*-values for SNPs, DNA methylation markers, and expression genes, respectively, we used a modified Fisher’s method [37] to combine the three *p*-values to one. In Fisher’s method, let *p*_*i*_ (*i* = 1, 2,…, *w*) be independent *p*-values obtained from *n* hypothesis tests. Under the null hypothesis that *p*-values follow a Uniform(0, 1) distribution, the combined test statistic is equal to $T=-2\sum_{i=1}^{w}ln(p_{i})$ that follows $\chi_{2w}^{2}$. However, within a gene, these *p*-values are correlated, thus the generalized Fisher’s method cannot be used directly. To address this issue, we consider a Satterthwaite approximation by approximating a scaled *T* statistic with a new chi-square distribution [38].

$$cT\approx \chi_{v}^{2},\;\mathrm{where}\;c=\frac{v}{E(T)},\;v=2\frac{{\left[E(T)\right]}^{2}}{Var(T)},$$

$$E(T)=E(-2\;\sum_{i=1}^{w}ln(p_{i}))=2w\;\mathrm{and}$$

$$Var(T)=var(-2\;\sum_{i=1}^{w}ln(p_{i}))=4w+2\sum_{i<j}cov(-2ln(p_{i}),\;-2ln(p_{j}))$$

where *w* = 3 for SNPs, DNA methylation markers, and expression genes. The covariance part takes the correlations of *p*-values into account and can be empirically estimated by perturbations. The perturbation details are described in the following section.

### Optimal test for the gene-level effects of SNPs, DNA methylation and RNA expression, using perturbations with *p*-values from the Omnibus-Fisher method as inputs

If the disease risk only depends on SNPs and the model with SNPs, DNA methylation markers, and expression genes is used, then the testing power will lose. Since in reality we do not know the underlying true disease model (e.g., only SNP effect, both SNP and RNA variant effects, or all SNP, RNA variant, and DNA methylation marker effects; totally 7 combinations), it is difficult to choose the correct model. Thus, it is desirable to develop a method accommodating all possible disease models to maximize power. This can be achieved by using the minimum *p*-value of all possible models (7 combinations) as a new test statistic. Then, perturbation can be used to calculate the final *p*-value.

The perturbation-based approach was described in Wu et al. [39]. For continuous phenotypes, with large *n*, under *H*_*0*_ the $(y-X\hat{\beta })/{\hat{\sigma }}_{E}$ are approximately standard normal. Then each ${Q=(y-X\hat{\beta })}^{\prime }GWG^{\prime }(y-X\hat{\beta })/{\hat{\sigma }}_{E}^{2}$ is essentially comprised of a vector of standard normal variables sandwiching a square matrix. Thus, we can perturb each Q by replacing $(y-X\hat{\beta })/{\hat{\sigma }}_{E}$ with a new, common vector of normal values to generate new score statistics. Following a similar procedure as described in Urrutia et al. [40]:

1. Calculate the *p*-values for SNPs (*G*), DNA methylation (*M*) and RNA expression (*E*) separately (i.e., $p_{G}^{\left(0\right)},p_{M}^{\left(0\right)}$, and $p_{E}^{\left(0\right)}$) by KM regression.

2. For *l* ∈ {*G*, *M* and *E*}, compute **Λ**_*l*_ = *diag*(*λ*_*l*,1_,⋯,*λ*_*l*,*ml*_), and **V**_*l*_ = [***v***_*l*,1_,⋯,***v***_*l*,*ml*_] where *λ*_*l*,1_ ≥ *λ*_*l*,2_ ≥⋯≥ λ_*l*,*ml*_ are the *ml* positive eigenvalues of ${P_{0l}}^{\frac{1}{2}}D_{l}W_{l}D_{l}^{\prime }{P_{0l}}^{\frac{1}{2}}$ with corresponding eigenvectors ***v***_*l*,1_,⋯,***v***_*l*,*ml*_, where **D**_*l*_ ∈ {omics data matrices **G**, **M** and **E**}. For example, the aforementioned ${P_{0}}^{\frac{1}{2}}GWG^{\prime }{P_{0}}^{\frac{1}{2}}$ is for *G*.

3. Generate $r^{\left(b\right)}={\left[r_{1}^{\left(b\right)},\cdots ,r_{n}^{\left(b\right)}\right]}^{\prime }$ with each $r_{j}^{\left(b\right)}∼N(0,1)$. This indicates that one subject has one $r_{j}^{\left(b\right)}$. If the subject has all *G*, *M* and *E*, the same $r_{j}^{\left(b\right)}$ will be used for *G*, *M* and *E*, respectively. Thus, whether *G*, *M* and *E* come from the same subjects or different subjects are considered.

4. For *l* ∈ {*G*, *M* and *E*}, rotate ***r***^(*b*)^ using the eigenvectors to generate $r_{l}^{\left(b\right)}=V_{l}^{\prime }r^{\left(b\right)}$.

5. Compute $Q_{l}^{\left(b\right)}=r_{l}^{{\left(b\right)}^{\prime }}\Lambda_{l}r_{l}^{\left(b\right)}$ for each *l* and obtain a corresponding *p*-value, $p_{l}^{\left(b\right)}$.

6. Repeat (3)-(5) *B* times to obtain $p_{G}^{\left(1\right)},p_{G}^{\left(2\right)},\cdots ,p_{G}^{\left(B\right)},\;p_{M}^{\left(1\right)},p_{M}^{\left(2\right)},\cdots ,p_{M}^{\left(B\right)}$ and $p_{E}^{\left(1\right)},p_{E}^{\left(2\right)},\cdots ,p_{E}^{\left(B\right)}$ for some large number *B*.

7. Calculate the covariance between *p*_*G*_, *p*_*M*_ and *p*_*E*_ by using $p_{G}^{\left(b\right)},p_{M}^{\left(b\right)}$, and $p_{E}^{\left(b\right)}$ for *b* ∈ {0, 1,…, *B*}.

8. Calculate the joint *p*-values of SNPs, DNA methylation and RNA expression (i.e., for *b* ∈ {0, 1,…, *B*}, $p_{GM}^{\left(b\right)},\;p_{GE}^{\left(b\right)},\;p_{ME}^{\left(b\right)}$, and $p_{GME}^{\left(b\right)}$) by Omnibus-Fisher considering *p*-values covariance.

9. For l* ∈ {*G*, *M*, *E*, *GM*, *GE*, *ME*, and *GME*}; *b* ∈ {0, 1,…, *B*}, set $p^{\left(b\right)}={min}_{1\leq l^{*}\leq L^{*}}p_{l^{*}}^{\left(b\right)}$.

10. The final *p*-value for significance is estimated as

$$p=B^{-1}\sum_{b=1}^{B}I(p^{\left(b\right)}\leq p^{\left(0\right)})$$

### Simulation studies

#### Pools of haplotypes, DNA methylation markers, and expression genes

For the pool of haplotypes, we simulated 10,000 haplotypes over a 200-kb region generated by the calibrated coalescent model [41], mimicking the European ancestry linkage disequilibrium (LD) structure. For the pool of DNA methylation, 689 samples with 8,557 CpG sites in chromosome 22 were extracted from an epigenome-wide association study of rheumatoid arthritis [42]. The DNA methylation data were generated using the Illumina 450K methylation array. For the pool of RNA expression, 142 samples with 327 genes in chromosome 22 were extracted from an expression continuous trait loci (eQTL) study [30]. The RNA expressions were assessed using the Illumina HT-12 microarray.

#### Simulation settings

We simulated two main settings: 1. G, M and E are independent with each other and 2. G and E are correlated, but independent with M. In each of the two main settings, we considered both continuous and binary traits. We evaluated the type I error rate by assuming the simulated phenotypes were independent with G, M and E, and evaluated the power performance by assuming the simulated phenotypes were dependent with (1) G; (2) G and M; or (3) G, M and E. Thus, we had the scenarios for G, M and E are independent: (1.1) continuous traits and no causal factors (denoted as null-c-ind), (1.2) continuous traits and G is causal (denoted as causal-G-c-ind), (1.3) continuous traits and G and M are causal (denoted as causal-GM-c-ind), (1.4) continuous traits and G, M and E are causal (denoted as causal-GME-c-ind), (1.5) binary traits and no causal factors (denoted as null-b-ind), (1.6) binary traits and G is causal (denoted as causal-G-b-ind), (1.7) binary traits and G and M are causal (denoted as causal-GM-b-ind), and (1.8) binary traits and G, M and E are causal (denoted as causal-GME-b-ind); and we had the scenarios for G and E are correlated: (2.1) continuous traits and no causal factors (denoted as null-c-cor), (2.2) continuous traits and G is causal (denoted as causal-G-c-cor), (2.3) continuous traits and G and M are causal (denoted as causal-GM-c-cor. When the causal SNPs in G are correlated with E, GM causal is similar to GME causal), (2.4) binary traits and no causal factors (denoted as null-b-cor), (2.5) binary traits and G is causal (denoted as causal-G-b-cor), and (2.6) binary traits and G and M are causal (denoted as causal-GM-b-cor). As shown in S2 Fig, we first simulated the scenarios of binary traits with causal factors.

#### Simulation of (1.6) causal-G-b-ind, (1.5) null-b-ind, (1.2) causal-G-c-ind and (1.1) null-c-ind

First, we simulated the scenario of (1.6) causal-G-b-ind. Following the flow in S3 Fig, we simulated 50 SNPs, 5 methylated sites and 1 RNA expression for one sample. Then, the phenotype for sample *i* was generated via the model:

$$logit\;P(y_{i}=1)=-2.94+0.001X_{i1}+0.001X_{i2}+\sum_{j=1}^{5}0.3G_{ij}$$

where -2.94 = log(0.05/0.95) indicates the disease prevalence is 0.05; X_*i*1_ is a continuous covariate generated from a normal distribution with a mean of 20 and a standard deviation of 1; X_*i*2_ is a binary covariate generated from a Bernoulli distribution with a probability of 0.5; G_*i*1_,G_*i*2_,…,G_*i*5_ are the genotypes of randomly selected causal SNPs. We saved the first 500 cases and 500 controls to form a dataset, and we simulated 1,000 such datasets (S3 Fig).

After obtaining (1.6), we permuted the binary traits 10 times for each of the 1,000 (1.6) datasets to get 10,000 datasets for the scenario of (1.5) null-b-ind (S2A Fig). Furthermore, we used the same G, M and E data as generated in (1.6) to simulate 1,000 sets of continuous phenotypes for (1.2) causal-G-c-ind (S2A Fig) via the following model:

$$y_{i}=0.001X_{i1}+0.001X_{i2}+\sum_{j=1}^{5}0.3G_{ij}+\varepsilon_{i}$$

where X_*i*1_, X_*i*2_ and G_*ij*_ are the same as described above; *ε*_*i*_ follows a standard normal distribution. We further permuted the continuous traits 10 times for each of the 1,000 (1.2) datasets to get 10,000 datasets for the scenario of (1.1) null-c-ind (S2A Fig).

#### Simulation of (1.7) causal-GM-b-ind and (1.3) causal-GM-c-ind

To simulate (1.7) causal-GM-b-ind, we followed a similar procedure to simulating (1.6) causal-G-b-ind via the model:

$$logit\;P(y_{i}=1)=-2.94+0.001X_{i1}+0.001X_{i2}+\sum_{j=1}^{5}0.2G_{ij}+\sum_{j=1}^{2}0.9M_{ij}$$

where X_*i*1_, X_*i*2_ and G_*ij*_ are the same as described above; M_*i*1_ and M_*i*2_ are the randomly selected causal methylated sites. We further used the same G, M and E data as generated in (1.7) to simulate 1,000 sets of continuous phenotypes for (1.3) causal-GM-c-ind (S2A Fig) via the following model:

$$y_{i}=0.001X_{i1}+0.001X_{i2}+\sum_{j=1}^{5}0.2G_{ij}+\sum_{j=1}^{2}0.9M_{ij}+\varepsilon_{i}$$

#### Simulation of (1.8) causal-GME-b-ind and (1.4) causal-GME-c-ind

Similarly, (1.8) causal-GME-b-ind and (1.4) causal-GME-c-ind were simulated via the following models for binary and continuous traits respectively:

$$\begin{array}{l} logit\;P(y_{i}=1)\\ =-2.94+0.001X_{i1}+0.001X_{i2}+\sum_{j=1}^{5}0.2G_{ij}+\sum_{j=1}^{2}0.9M_{ij}+1(E_{i}-7.45)\\ y_{i}=0.001X_{i1}+0.001X_{i2}+\sum_{j=1}^{5}0.2G_{ij}+\sum_{j=1}^{2}0.9M_{ij}+1(E_{i}-7.45)+\varepsilon_{i} \end{array}$$

where X_*i*1_, X_*i*2_, G_*ij*_ and M_*ij*_ are the same as described above; E_*i*_ is the RNA expression and 7.45 is the global mean of RNA expression in the pool.

Finally, all the scenarios in the setting of assuming G and E are correlated (i.e., (2.1) ~ (2.6)) were simulated in the analogous ways to the first setting and used the same models. The only difference is that one causal SNP in the gene is correlated with its RNA expression:

$$E_{i}^{*}=E_{i}+G_{i1}$$

where E_*i*_ is the aforementioned RNA expression used in the setting of G, M and E independent, G_*i*1_ is the first causal SNP in the simulated gene, and $E_{i}^{*}$ is the RNA expression used in the setting of G and E correlated. We compared the optimal test (optimal Omnibus-Fisher) of G, M and E with the Fisher’s method combing G, M and E either considering covariance (Omnibus-Fisher) or not (usual Fisher’s method).

### Studies of childhood asthma in Puerto Ricans

#### Genetic variants

Subject recruitment and study procedures have been described in detail [43, 44]. In brief, 449 children ages 6–14 yrs were recruited from schools in Hartford (CT) from 09/03 to 07/08. From 03/09 to 06/10, 678 children ages 6–14 yrs were recruited in San Juan (Puerto Rico) using a multistage probabilistic sampling design [45]. At each study site, there were no significant differences in age, sex, or area of residence between eligible children whose parents did or did not agree to participate. At both sites, the main recruitment tool was a screening questionnaire on the child’s respiratory health and PR ancestry. We selected case children with physician-diagnosed asthma and wheezing in the prior year. All children had four PR grandparents. The genotypes were coded as 0, 1 and 2 indicating the number of rare allele copies. The imputation was performed on Michigan Imputation Server [46] with HRC r1.1 2016 [47] as the reference genome.

#### Epigenetic variants

Children with and without asthma (aged 9–20 years) were recruited in San Juan (PR) from February 2014 to May 2017, using a similar approach to that used in a previous study [48]. Whole-genome methylation assays were performed using HumanMethylation450 BeadChips (Illumina, San Diego, CA), and M-values were used in all downstream analyses. RNA-Seq was performed with the Illumina NextSeq 500 platform, paired-end reads at 75 cycles, and 80M reads/sample; reads were aligned to reference human genome (hg19) [49] and TPM (Transcripts Per Kilobase Million) were used as proxy for gene expression level.

**Fig 5:**
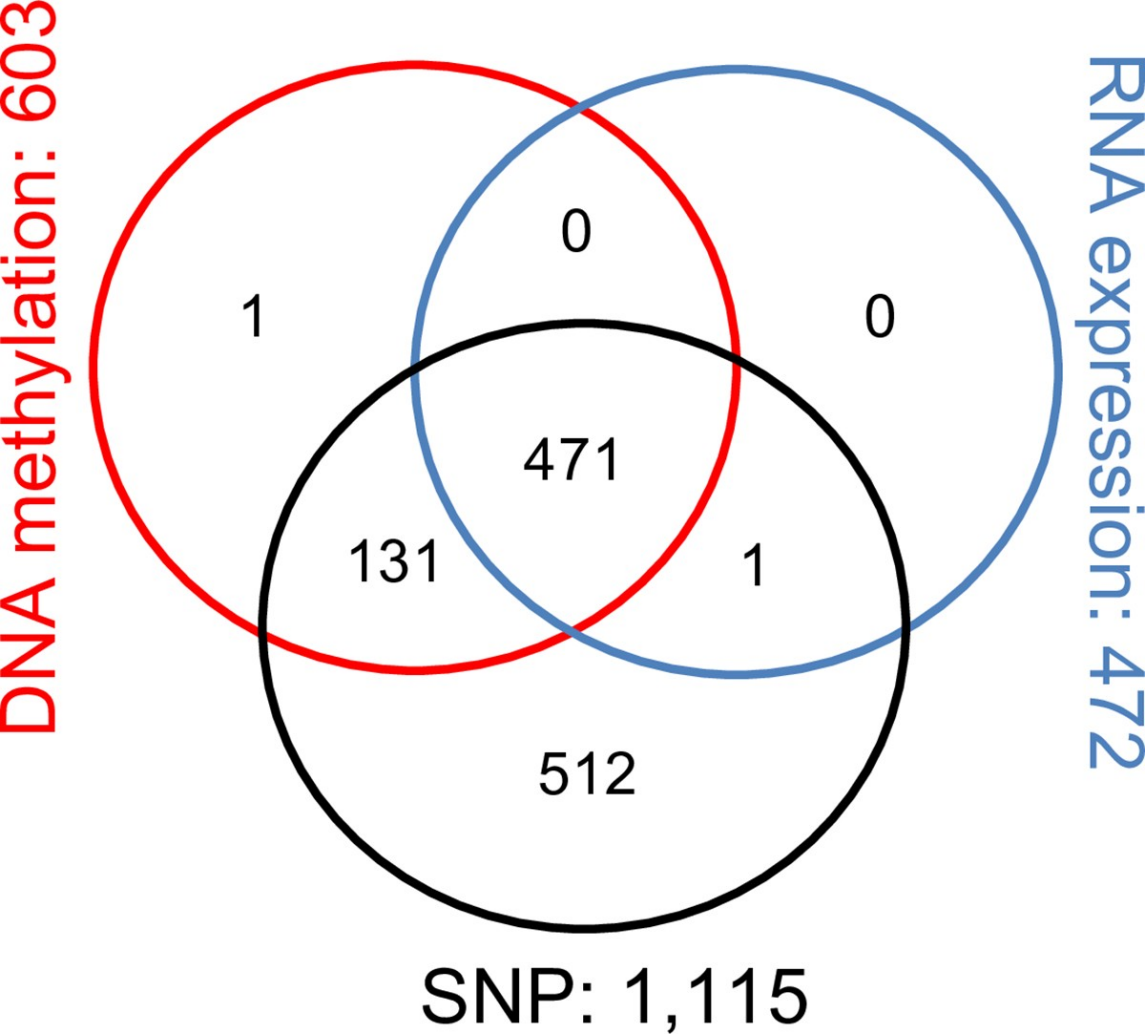
The omics data distribution in the Puerto Rican childhood asthma dataset.

In summary, we acquired multiple-dimensional data from microarray platforms for SNP genotyping and DNA methylation from white blood cells (WBCs), and RNA sequence data from WBCs in PR children. 10,994,111 imputed and genotyped SNPs with no missing genotypes were selected and they were assigned to 22,332 genes. 368,529 methylated sites with M values were selected and they were assigned to 20,596 genes. 16,188 genes with average TPM value greater than 0.05 were included for RNA expression. In the final analysis, we used the 14,808 genes with all SNP, DNA methylation and RNA expression information, and 1,116 subjects. Note that 471 out of the 1,116 subjects have all three types of omics data (**Fig 5**).

## Supporting information

S1 Table Simulated Type I error rates based on 100,000 datasets. (DOCX)

S1 Fig The p-value correlation for each gene across the whole genome. (A) between SNPs and DNA methylation markers, (B) between SNPs and expression genes, and (C) between DNA methylation markers and expression genes.
(PDF)

S2 Fig Simulation scenarios. (A) SNPs, DNA methylation and RNA expression are independent; (B) SNPs and RNA expression are correlated.
(PDF)

S3 Fig Simulation details of the scenario of (1.6) causal-G-b-ind. (PDF)

## References

[1] Manolio TA, Collins FS, Cox NJ, Goldstein DB, Hindorff LA, Hunter DJ, et al Finding the missing heritability of complex diseases. Nature. 2009;461(7265):747–53. 10.1038/nature08494 19812666 PMC2831613

[2] Phan JH, Quo CF, Cheng C, Wang MD. Multiscale integration of -omic, imaging, and clinical data in biomedical informatics. IEEE Rev Biomed Eng. 2012;5:74–87. 10.1109/RBME.2012.2212427 . 23231990 PMC5859561

[3] Song R, Huang J, Ma S. Integrative prescreening in analysis of multiple cancer genomic studies. BMC Bioinformatics. 2012;13:168 10.1186/1471-2105-13-168 22799431 PMC3436748

[4] Bersanelli M, Mosca E, Remondini D, Giampieri E, Sala C, Castellani G, et al Methods for the integration of multi-omics data: mathematical aspects. BMC Bioinformatics. 2016;17 Suppl 2:15 10.1186/s12859-015-0857-9 26821531 PMC4959355

[5] Li B, Leal SM. Methods for detecting associations with rare variants for common diseases: application to analysis of sequence data. Am J Hum Genet. 2008;83(3):311–21. 10.1016/j.ajhg.2008.06.024 18691683 PMC2842185

[6] Madsen BE, Browning SR. A groupwise association test for rare mutations using a weighted sum statistic. PLoS genetics. 2009;5(2):e1000384 10.1371/journal.pgen.1000384 19214210 PMC2633048

[7] Morgenthaler S, Thilly WG. A strategy to discover genes that carry multi-allelic or mono-allelic risk for common diseases: a cohort allelic sums test (CAST). Mutation research. 2007;615(1–2):28–56. 10.1016/j.mrfmmm.2006.09.003 . 17101154

[8] Li B, Leal SM. Discovery of rare variants via sequencing: implications for the design of complex trait association studies. PLoS genetics. 2009;5(5):e1000481 10.1371/journal.pgen.1000481 19436704 PMC2674213

[9] Price AL, Kryukov GV, de Bakker PI, Purcell SM, Staples J, Wei LJ, et al Pooled association tests for rare variants in exon-resequencing studies. American journal of human genetics. 2010;86(6):832–8. 10.1016/j.ajhg.2010.04.005 20471002 PMC3032073

[10] Han F, Pan W. A data-adaptive sum test for disease association with multiple common or rare variants. Human heredity. 2010;70(1):42–54. 10.1159/000288704 20413981 PMC2912645

[11] Morris AP, Zeggini E. An evaluation of statistical approaches to rare variant analysis in genetic association studies. Genetic epidemiology. 2010;34(2):188–93. 10.1002/gepi.20450 19810025 PMC2962811

[12] Lin WY, Yi N, Zhi D, Zhang K, Gao G, Tiwari HK, et al Haplotype-based methods for detecting uncommon causal variants with common SNPs. Genetic epidemiology. 2012;36(6):572–82. 10.1002/gepi.21650 22706849 PMC3513398

[13] Lin WY, Yi N, Lou XY, Zhi D, Zhang K, Gao G, et al Haplotype kernel association test as a powerful method to identify chromosomal regions harboring uncommon causal variants. Genetic epidemiology. 2013;37(6):560–70. 10.1002/gepi.21740 . 23740760 PMC4116485

[14] Lin WY, Lou XY, Gao G, Liu N. Rare Variant Association Testing by Adaptive Combination of P-values. PloS one. 2014;9(1):e85728 10.1371/journal.pone.0085728 24454922 PMC3893264

[15] Yan Q, Tiwari HK, Yi N, Lin WY, Gao G, Lou XY, et al Kernel-machine testing coupled with a rank-truncation method for genetic pathway analysis. Genetic epidemiology. 2014;38(5):447–56. 10.1002/gepi.21813 24849109 PMC4073214

[16] Wu MC, Kraft P, Epstein MP, Taylor DM, Chanock SJ, Hunter DJ, et al Powerful SNP-set analysis for case-control genome-wide association studies. American journal of human genetics. 2010;86(6):929–42. 10.1016/j.ajhg.2010.05.002 20560208 PMC3032061

[17] Wu MC, Lee S, Cai T, Li Y, Boehnke M, Lin X. Rare-variant association testing for sequencing data with the sequence kernel association test. Am J Hum Genet. 2011;89(1):82–93. 10.1016/j.ajhg.2011.05.029 21737059 PMC3135811

[18] Torgerson DG, Ampleford EJ, Chiu GY, Gauderman WJ, Gignoux CR, Graves PE, et al Meta-analysis of genome-wide association studies of asthma in ethnically diverse North American populations. Nat Genet. 2011;43(9):887–92. 10.1038/ng.888 21804549 PMC3445408

[19] Moffatt MF, Gut IG, Demenais F, Strachan DP, Bouzigon E, Heath S, et al A large-scale, consortium-based genomewide association study of asthma. N Engl J Med. 2010;363(13):1211–21. 10.1056/NEJMoa0906312 20860503 PMC4260321

[20] Bouzigon E, Corda E, Aschard H, Dizier MH, Boland A, Bousquet J, et al Effect of 17q21 variants and smoking exposure in early-onset asthma. N Engl J Med. 2008;359(19):1985–94. Epub 2008/10/17. NEJMoa0806604 [pii] 10.1056/NEJMoa0806604 . 18923164

[21] Galanter J, Choudhry S, Eng C, Nazario S, Rodriguez-Santana JR, Casal J, et al ORMDL3 gene is associated with asthma in three ethnically diverse populations. American journal of respiratory and critical care medicine. 2008;177(11):1194–200. 10.1164/rccm.200711-1644OC 18310477 PMC2408437

[22] Halapi E, Gudbjartsson DF, Jonsdottir GM, Bjornsdottir US, Thorleifsson G, Helgadottir H, et al A sequence variant on 17q21 is associated with age at onset and severity of asthma. European journal of human genetics: EJHG. 2010;18(8):902–8. 10.1038/ejhg.2010.38 20372189 PMC2987388

[23] Leung TF, Sy HY, Ng MC, Chan IH, Wong GW, Tang NL, et al Asthma and atopy are associated with chromosome 17q21 markers in Chinese children. Allergy. 2009;64(4):621–8. 10.1111/j.1398-9995.2008.01873.x . 19175592

[24] Madore AM, Tremblay K, Hudson TJ, Laprise C. Replication of an association between 17q21 SNPs and asthma in a French-Canadian familial collection. Human genetics. 2008;123(1):93–5. 10.1007/s00439-007-0444-x . 17992541

[25] Sleiman PM, Annaiah K, Imielinski M, Bradfield JP, Kim CE, Frackelton EC, et al ORMDL3 variants associated with asthma susceptibility in North Americans of European ancestry. The Journal of allergy and clinical immunology. 2008;122(6):1225–7. 10.1016/j.jaci.2008.06.041 . 18760456

[26] Tavendale R, Macgregor DF, Mukhopadhyay S, Palmer CN. A polymorphism controlling ORMDL3 expression is associated with asthma that is poorly controlled by current medications. The Journal of allergy and clinical immunology. 2008;121(4):860–3. 10.1016/j.jaci.2008.01.015 . 18395550

[27] Bisgaard H, Bonnelykke K, Sleiman PM, Brasholt M, Chawes B, Kreiner-Moller E, et al Chromosome 17q21 gene variants are associated with asthma and exacerbations but not atopy in early childhood. American journal of respiratory and critical care medicine. 2009;179(3):179–85. 10.1164/rccm.200809-1436OC . 19029000

[28] Galanter JM, Gignoux CR, Torgerson DG, Roth LA, Eng C, Oh SS, et al Genome-wide association study and admixture mapping identify different asthma-associated loci in Latinos: the Genes-environments & Admixture in Latino Americans study. The Journal of allergy and clinical immunology. 2014;134(2):295–305. 10.1016/j.jaci.2013.08.055 24406073 PMC4085159

[29] Yan Q, Brehm J, Pino-Yanes M, Forno E, Lin J, Oh SS, et al A meta-analysis of genome-wide association studies of asthma in Puerto Ricans. Eur Respir J. 2017;49(5). 10.1183/13993003.01505-2016 . 28461288 PMC5527708

[30] Chen W, Brehm JM, Lin J, Wang T, Forno E, Acosta-Perez E, et al Expression quantitative trait loci (eQTL) mapping in Puerto Rican children. PloS one. 2015;10(3):e0122464 10.1371/journal.pone.0122464 25816334 PMC4376710

[31] Kwee LC, Liu D, Lin X, Ghosh D, Epstein MP. A powerful and flexible multilocus association test for quantitative traits. American journal of human genetics. 2008;82(2):386–97. 10.1016/j.ajhg.2007.10.010 18252219 PMC2664991

[32] Liu D, Lin X, Ghosh D. Semiparametric regression of multidimensional genetic pathway data: least-squares kernel machines and linear mixed models. Biometrics. 2007;63(4):1079–88. 10.1111/j.1541-0420.2007.00799.x 18078480 PMC2665800

[33] Zhang D, Lin X. Hypothesis testing in semiparametric additive mixed models. Biostatistics. 2003;4(1):57–74. 10.1093/biostatistics/4.1.57 . 12925330

[34] Chen H, Meigs JB, Dupuis J. Sequence kernel association test for quantitative traits in family samples. Genet Epidemiol. 2013;37(2):196–204. 10.1002/gepi.21703 23280576 PMC3642218

[35] Davies R. The distribution of a linear combination of chi-square random variables. J R Stat Soc Ser C Appl Stat. 1980;29:323–33.

[36] Kuonen D. Saddlepoint approximations for distributions of quadratic forms in normal variables. Biometrika. 1999;86:929–35.

[37] Fisher RA. Statistical Methods for Research Workers: Oliver and Boyd (Edinburgh); 1925.

[38] Li SY, Williams BL, Cui YH. A combined p-value approach to infer pathway regulations in eQTL mapping. Stat Interface. 2011;4(3):389–401. WOS:000298000300013.

[39] Wu MC, Maity A, Lee S, Simmons EM, Harmon QE, Lin X, et al Kernel machine SNP-set testing under multiple candidate kernels. Genet Epidemiol. 2013;37(3):267–75. Epub 2013/03/09. 10.1002/gepi.21715 23471868 PMC3769109

[40] Urrutia E, Lee S, Maity A, Zhao N, Shen J, Li Y, et al Rare variant testing across methods and thresholds using the multi-kernel sequence kernel association test (MK-SKAT). Stat Interface. 2015;8(4):495–505. Epub 2016/01/08. 10.4310/SII.2015.v8.n4.a8 26740853 PMC4698916

[41] Schaffner SF, Foo C, Gabriel S, Reich D, Daly MJ, Altshuler D. Calibrating a coalescent simulation of human genome sequence variation. Genome research. 2005;15(11):1576–83. 10.1101/gr.3709305 16251467 PMC1310645

[42] Liu Y, Aryee MJ, Padyukov L, Fallin MD, Hesselberg E, Runarsson A, et al Epigenome-wide association data implicate DNA methylation as an intermediary of genetic risk in rheumatoid arthritis. Nat Biotechnol. 2013;31(2):142–7. 10.1038/nbt.2487 23334450 PMC3598632

[43] Brehm JM, Acosta-Perez E, Klei L, Roeder K, Barmada MM, Boutaoui N, et al African ancestry and lung function in Puerto Rican children. J Allergy Clin Immunol. 2012;129(6):1484–90 e6. 10.1016/j.jaci.2012.03.035 22560959 PMC3367038

[44] Forno E, Cloutier MM, Datta S, Paul K, Sylvia J, Calvert D, et al Mouse allergen, lung function, and atopy in Puerto Rican children. PloS one. 2012;7(7):e40383 10.1371/journal.pone.0040383 22815744 PMC3398035

[45] Bird HR, Canino GJ, Davies M, Duarte CS, Febo V, Ramirez R, et al A study of disruptive behavior disorders in Puerto Rican youth: I. Background, design, and survey methods. J Am Acad Child Adolesc Psychiatry. 2006;45(9):1032–41. 10.1097/01.chi.0000227878.58027.3d . 16926610

[46] Das S, Forer L, Schonherr S, Sidore C, Locke AE, Kwong A, et al Next-generation genotype imputation service and methods. Nat Genet. 2016;48(10):1284–7. Epub 2016/08/30. 10.1038/ng.3656 27571263 PMC5157836

[47] McCarthy S, Das S, Kretzschmar W, Delaneau O, Wood AR, Teumer A, et al A reference panel of 64,976 haplotypes for genotype imputation. Nat Genet. 2016;48(10):1279–83. Epub 2016/08/23. 10.1038/ng.3643 27548312 PMC5388176

[48] Forno E WT, Qi C, Yan Q, Xu C, Boutaoui N, Han Y, Weeks D, Jiang Y, Rosser F, Vonk J, Brouwer S, Acosta-Pérez E, Colón-Semidey A, Alvarez M, Canino G, Koppelman G, Chen W, Celedón JC DNA methylation in nasal epithelium, atopy, and atopic asthma in children: a genome-wide study. 2018. Epub Ahead of print 10.1016/S2213-2600(18)30466-1 PMC6441380 30584054

[49] Dobin A, Davis CA, Schlesinger F, Drenkow J, Zaleski C, Jha S, et al STAR: ultrafast universal RNA-seq aligner. Bioinformatics. 2013;29(1):15–21. 10.1093/bioinformatics/bts635 23104886 PMC3530905

