# Supplementary material for: An integrative association method for omics data based on a modified Fisher’s method with application to childhood asthma
Source: PLoS Genet. 2019 May 7;15(5):e1008142. doi: 10.1371/journal.pgen.1008142 (PMC6524814; doi:10.1371/journal.pgen.1008142)
Supplement: S1 Table — (DOCX) [file pgen.1008142.s001.docx]

Table S1. Simulated Type I error rates based on 100,000 datasets

| **Significance level** | **Optimal Omnibus-Fisher** | **Omnibus-Fisher** | **Usual Fisher** |
| --- | --- | --- | --- |
| *Independent G, M and E with binary traits* | | | |
| 0.05 | 0.04920 | 0.04965 | 0.04983 |
| 0.01 | 0.00946 | 0.01000 | 0.01008 |
| 0.001 | 0.00120 | 0.00113 | 0.00114 |
| 0.0001 | 0.00007 | 0.00010 | 0.00010 |
| *Independent G, M and E with continuous traits* | | | |
| 0.05 | 0.04965 | 0.04977 | 0.04999 |
| 0.01 | 0.00934 | 0.00982 | 0.01000 |
| 0.001 | 0.00085 | 0.00086 | 0.00090 |
| 0.0001 | 0.00011 | 0.00010 | 0.00010 |
| *G and E correlated with binary traits* | | | |
| 0.05 | 0.04913 | 0.04981 | 0.06342 |
| 0.01 | 0.00955 | 0.01092 | 0.01722 |
| 0.001 | 0.00101 | 0.00134 | 0.00331 |
| 0.0001 | 0.00008 | 0.00018 | 0.00066 |
| *G and E correlated with continuous traits* | | | |
| 0.05 | 0.04965 | 0.04977 | 0.06398 |
| 0.01 | 0.00934 | 0.00982 | 0.01786 |
| 0.001 | 0.00085 | 0.00086 | 0.00329 |
| 0.0001 | 0.00011 | 0.00010 | 0.00060 |
