# Supplementary material for: An integrative association method for omics data based on a modified Fisher’s method with application to childhood asthma
Source: PLoS Genet. 2019 May 7;15(5):e1008142. doi: 10.1371/journal.pgen.1008142 (PMC6524814; doi:10.1371/journal.pgen.1008142)
Supplement: S1 Fig — (A) between SNPs and DNA methylation markers, (B) between SNPs and expression genes, and (C) between DNA methylation markers and expression genes. (PDF) [file pgen.1008142.s002.pdf]

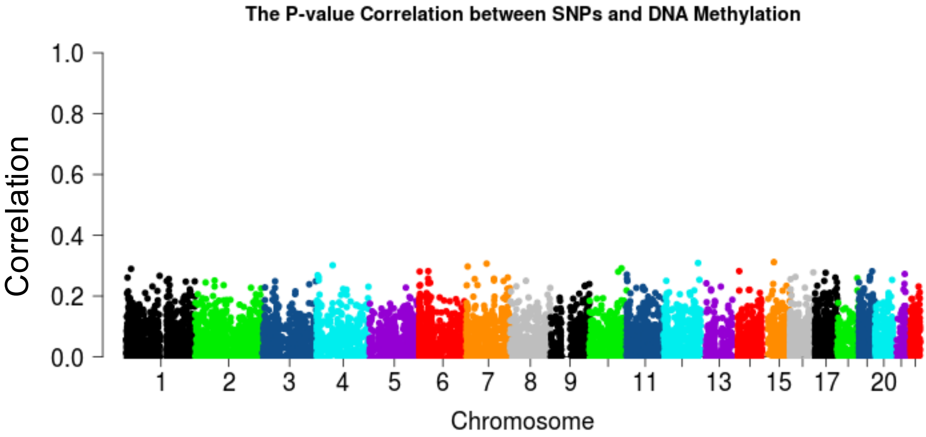
The P-value Correlation between SNPs and DNA Methylation
Correlation
0.0
0.2
0.4
0.6
0.8
1.0
1 2 3 4 5 6 7 8 9 11 13 15 17 20
Chromosome

(A)

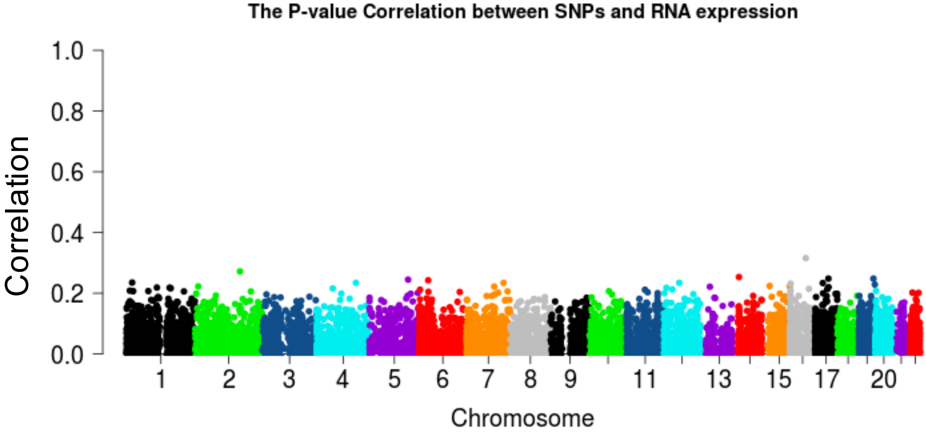
The P-value Correlation between SNPs and RNA expression
Correlation
0.0
0.2
0.4
0.6
0.8
1.0
1 2 3 4 5 6 7 8 9 11 13 15 17 20
Chromosome

(B)

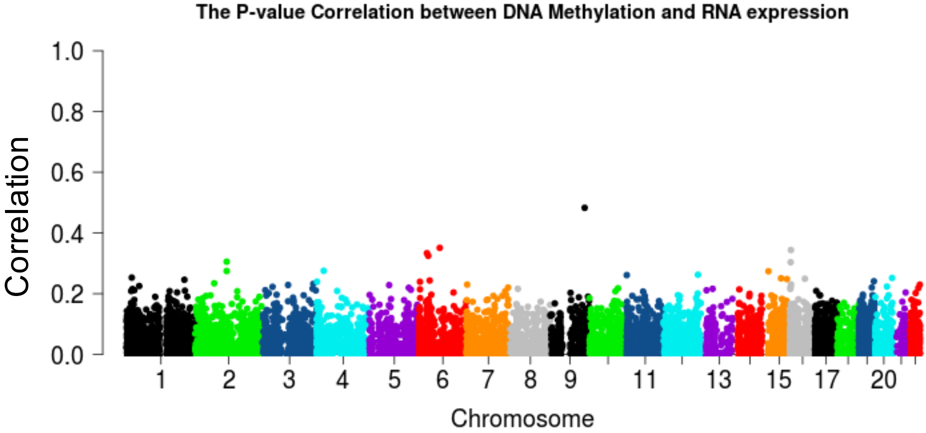
The P-value Correlation between DNA Methylation and RNA expression
Correlation
0.0
0.2
0.4
0.6
0.8
1.0
1 2 3 4 5 6 7 8 9 11 13 15 17 20
Chromosome

(C)
