# Supplementary material for: An integrative association method for omics data based on a modified Fisher’s method with application to childhood asthma
Source: PLoS Genet. 2019 May 7;15(5):e1008142. doi: 10.1371/journal.pgen.1008142 (PMC6524814; doi:10.1371/journal.pgen.1008142)
Supplement: S2 Fig — (A) SNPs, DNA methylation and RNA expression are independent; (B) SNPs and RNA expression are correlated. (PDF) [file pgen.1008142.s003.pdf]

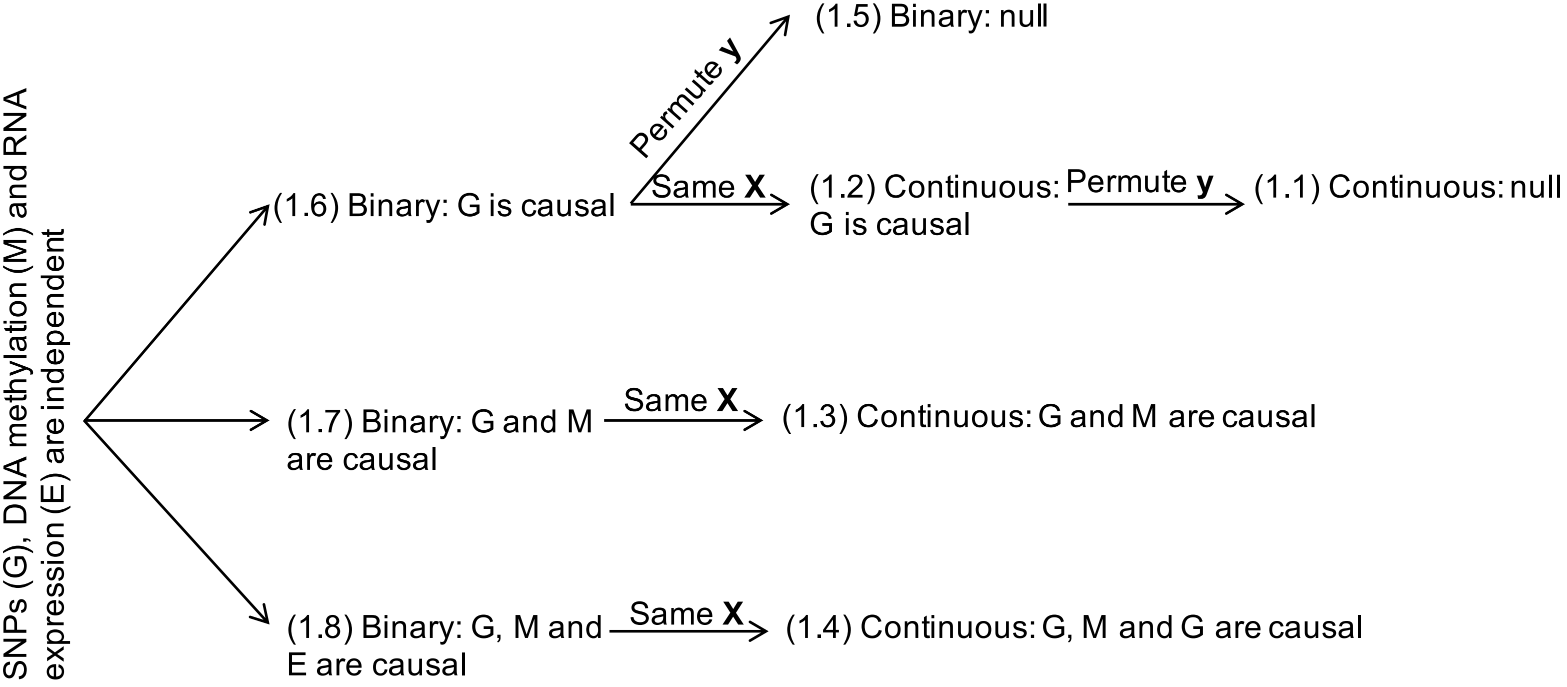
SNPs (G), DNA methylation (M) and RNA expression (E) are independent
(1.6) Binary: G is causal
Permute **y**
(1.5) Binary: null
Same **X**
(1.2) Continuous: G is causal
Permute **y**
(1.1) Continuous: null
(1.7) Binary: G and M are causal
Same **X**
(1.3) Continuous: G and M are causal
(1.8) Binary: G, M and E are causal
Same **X**
(1.4) Continuous: G, M and G are causal

(A)

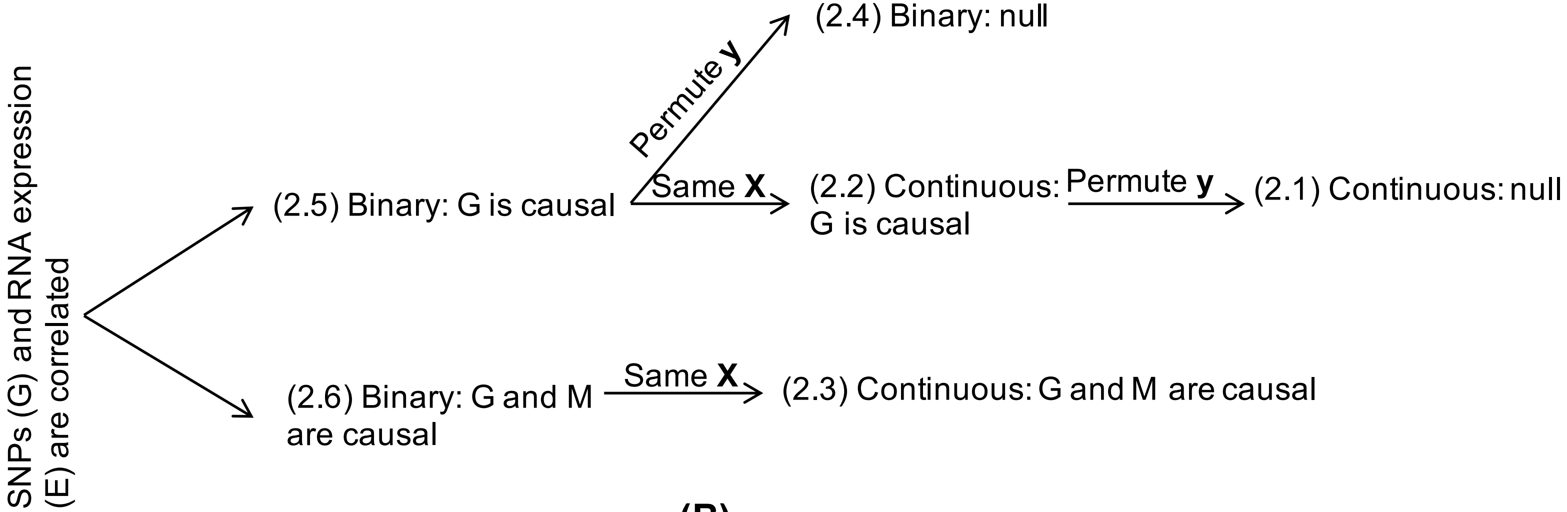
SNPs (G) and RNA expression (E) are correlated
(2.5) Binary: G is causal
Permute **y**
(2.4) Binary: null
Same **X**
(2.2) Continuous: G is causal
Permute **y**
(2.1) Continuous: null
(2.6) Binary: G and M are causal
Same **X**
(2.3) Continuous: G and M are causal

(B)
