# Supplementary material for: An integrative association method for omics data based on a modified Fisher’s method with application to childhood asthma
Source: PLoS Genet. 2019 May 7;15(5):e1008142. doi: 10.1371/journal.pgen.1008142 (PMC6524814; doi:10.1371/journal.pgen.1008142)
Supplement: S3 Fig — (PDF) [file pgen.1008142.s004.pdf]

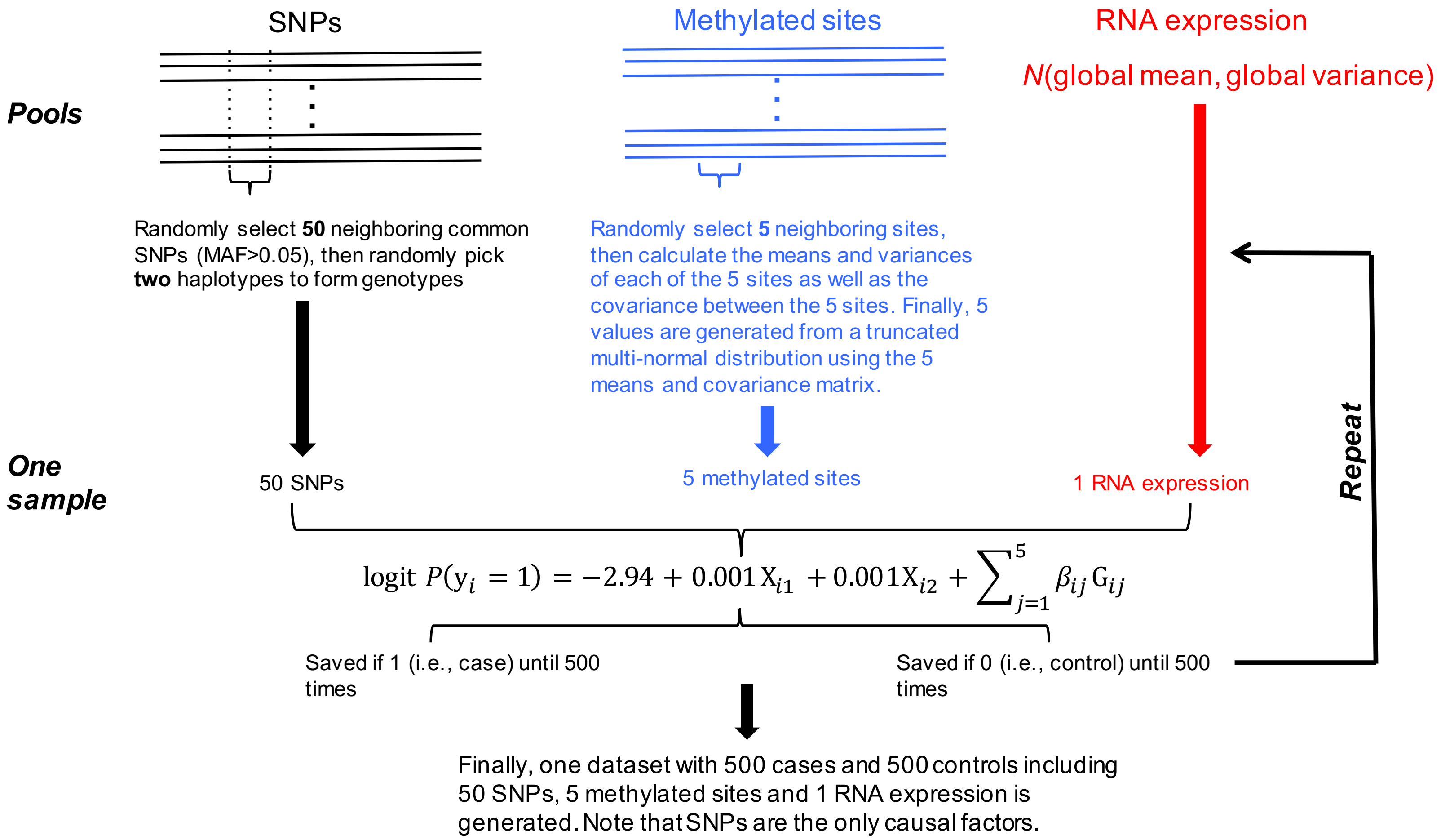
SNPs
Methylated sites
RNA expression
Pools
N(global mean, global variance)
Randomly select 50 neighboring common SNPs (MAF>0.05), then randomly pick two haplotypes to form genotypes
Randomly select 5 neighboring sites, then calculate the means and variances of each of the 5 sites as well as the covariance between the 5 sites. Finally, 5 values are generated from a truncated multi-normal distribution using the 5 means and covariance matrix.
Repeat
One sample
50 SNPs
5 methylated sites
1 RNA expression
$\text{logit } P(y_i = 1) = -2.94 + 0.001X_{i1} + 0.001X_{i2} + \sum_{j=1}^{5} \beta_{ij} G_{ij}$
Saved if 1 (i.e., case) until 500 times
Saved if 0 (i.e., control) until 500 times
Finally, one dataset with 500 cases and 500 controls including 50 SNPs, 5 methylated sites and 1 RNA expression is generated. Note that SNPs are the only causal factors.
